# Effects of Sensor Types and Angular Velocity Computational Methods in Field Measurements of Occupational Upper Arm and Trunk Postures and Movements

**DOI:** 10.3390/s21165527

**Published:** 2021-08-17

**Authors:** Xuelong Fan, Carl Mikael Lind, Ida-Märta Rhen, Mikael Forsman

**Affiliations:** 1 IMM Institute of Environmental Medicine, Karolinska Institutet, SE-171 77 Stockholm, Sweden; xuelong.fan@ki.se (X.F.); ida-marta.rhen@ki.se (I.-M.R.); miforsm@kth.se (M.F.); 2 Division of Ergonomics, School of Engineering Sciences in Chemistry, Biotechnology and Health, KTH Royal Institute of Technology, Hälsovägen 11C, SE-141 57 Huddinge, Sweden; 3 Centre for Occupational and Environmental Medicine, Stockholm County Council, SE-113 65 Stockholm, Sweden; 4 Department of Industrial and Materials Science, Chalmers University of Technology, SE-412 96 Gothenburg, Sweden

**Keywords:** inertial measurement unit, field measurement, accelerometer, sensor fusion, kinematics, threshold limit value, ergonomics, workload, biomechanics, musculoskeletal disorders

## Abstract

Accelerometer-based inclinometers have dominated kinematic measurements in previous field studies, while the use of inertial measurement units that additionally include gyroscopes is rapidly increasing. Recent laboratory studies suggest that these two sensor types and the two commonly used angular velocity computational methods may produce substantially different results. The aim of this study was, therefore, to evaluate the effects of sensor types and angular velocity computational methods on the measures of work postures and movements in a real occupational setting. Half-workday recordings of arm and trunk postures, and movements from 38 warehouse workers were compared using two sensor types: accelerometers versus accelerometers with gyroscopes—and using two angular velocity computational methods, i.e., inclination velocity versus generalized velocity. The results showed an overall small difference (<2° and value independent) for posture percentiles between the two sensor types, but substantial differences in movement percentiles both between the sensor types and between the angular computational methods. For example, the group mean of the 50th percentiles were for accelerometers: 71°/s (generalized velocity) and 33°/s (inclination velocity)—and for accelerometers with gyroscopes: 31°/s (generalized velocity) and 16°/s (inclination velocity). The significant effects of sensor types and angular computational methods on angular velocity measures in field work are important in inter-study comparisons and in comparisons to recommended threshold limit values.

## 1. Introduction

Work-related diseases and disorders are a global concern that effect societies, organizations, and individuals. Pain and disorders in the musculoskeletal system are one of the leading causes of quality-adjusted life-years lost due to ill health and disability [1]. On the society level, the attributed costs of occupational injuries and diseases have been estimated to be 3.9% of the gross domestic product globally, and 3.3% within the EU [2]. The major work-related risk factors for musculoskeletal disorders (MSDs) include awkward postures, frequent movements, forceful exertion postures, heavy manual handling [3,4,5,6,7] and psychosocial factors such as job strain [8]. A large proportion of workers are frequently exposed to these risk factors [9]. The specific risk factors associated with shoulder disorders include extended time with arms in elevated postures [4,10,11,12] and high arm movement velocities [13,14]. For the low back, peak- and cumulative spinal load [15,16], cumulative rest time [17], and high trunk movement velocities [18,19,20] have been associated with low back pain (LBP) or a decline in low back function. While several studies have reported an association between prolonged time in trunk flexion or in trunk axial rotation, and LBP or low back disorders [3,21], others report no significant associations or conflicting results [22,23,24], or negative associations [25]. To compare different studies, and to possibly find the reasons for the different results, it is important to know how the different technologies and methods that are used to measure and analyze the exposures may be compared.

In the last few decades, assessments of biomechanical exposures have predominantly relied on measurements obtained by self-reports and observation-based tools. While both can collect a large range of exposures, they often provide crude estimations of biomechanical exposures. They also often have low reliability and validity for ratings of movements and for upper-limb postures [26,27,28,29,30]. Therefore, technical instruments, such as accelerometer-based inclinometers, may be used to improve the accuracy and precision of these measurements [31]. Accelerometer-based inclinometers are increasingly becoming more accessible, easy-to-use, and less expensive [32], and are thus a feasible option for non-research applications. Consequently, during the last two decades (see Table 1), an increasing number of studies have utilized tri-axial accelerometers to monitor work postures, movements, and physical activity. Threshold limits for upper arm postures and velocities to minimize the risks of developing MSDs have also been drawn from a pool of such studies [14,33].

While accelerometers can produce accurate and precise measurements of inclination angles and angular velocities at low velocities [34,35], they are susceptible to the introduction of errors at high movement velocities [36,37,38,39,40]. Laboratory-based studies with standard movements and simulated work tasks have indicated that these errors can be substantially reduced by additionally utilizing gyroscopes in inertial measurement units (IMUs) [37,40,41]. Specifically, by using linear Kalman filter to integrate accelerometers and gyroscopes, the root mean square of error (RMSE) of inclination angles during simple movements can be maintained under 2° (versus less than 11° by using only accelerometers), and that of angular velocities can be under 10°/s (versus less than 80°/s by using only accelerometers) [37]. The extent to which the differences between accelerometer-based measurement and IMU-based measurement have an impact on measurements in a working population have, according to the best of our knowledge, not previously been investigated. This information is needed to ensure correct comparisons of exposure estimates obtained in studies where different sensor types have been used. The number of studies using integrated sensors in IMUs increased after 2016 (Table 1). Given the increase in the use of IMUs, this information is also needed when comparing new IMU-based measurements as in, e.g., Lind et al. [42], Lind et al. [43], to the recommended threshold limit values based on accelerometer-based measurements [14]. Moreover, this information can also be used for merging results from older and more recent studies to increase the precision on the associations between inclinations and angular velocities of arms and trunk, and MSDs.

Among studies with technical instruments, postural angles and movement velocities have been defined differently in body parts for practical reasons. For the trunk, it can be expressed as sagittal inclination angle and lateral inclination angle. The sagittal inclination angle (i.e., forward angle or flexion angle) is usually of higher amplitude than the side-way ditto [44,45], and it is normally the only angle reported for the trunk [46]. The angular velocity for the trunk movement has commonly been computed as the absolute value of the derivative of the sagittal inclination angle, namely sagittal inclination velocity. 

For the arms, the posture is normally described as inclination or elevation angle; that is, “the angle between the upper arm vector and the vertical line” [47]. Two different angular velocity computational methods have been used to estimate arm movements. The first method is the inclination (or elevation) velocity, which is computed using the derivative of the inclination angle. The second, i.e., the generalized velocity [34], includes the sensor rotation around all its axes to compute the angular velocity and is calculated as the angle travelled on the unit sphere per time unit. Contrary to the inclination velocity, the generalized velocity also includes axial arm rotation, which in theory should produce higher velocity values. This difference complicates comparisons between studies that have different angular velocity computational methods (Table 1). The ratio between these two velocities has, according to the best of our knowledge, not previously been examined in field studies. Without this information, it is difficult to conduct inter-study comparisons. It is also difficult to conduct comparisons of study results with recommended threshold limit values that are based on certain computational procedures, such as Balogh et al. [14] and Arvidsson et al. [48], who recommended that the full workday median arm (generalized) velocity should be below 60°/s. 

As shown in Table 1, many studies have been carried out that have only measured generalized arm velocity with accelerometers. However, recent studies have begun to use accelerometers with gyroscopes and inclination velocity. Therefore, both sensor type and angular velocity computational method comparisons with field data are needed.

**Table 1:** Summary (non-exhaustive) of articles from 2000 to date regarding the measurements of kinematics of arm and trunk by accelerometers or IMUs. Bold reference numbers indicate that the study, in addition of arm posture, also includes trunk posture (and/or velocity).

References	Sensor Type	Angular Velocity Computational Method	Posture	Type of Work	Publishing Year	Country of Data Collection
[12,31,38,49,50,51,52,53,54,55,56,57,58]	accelerometers only	-	arm, arm and trunk [31,52,54,56,58]	field	2004–2020	Sweden, Denmark, Norway, Brazil, North America, Australia
[59,60,61,62]	accelerometers only	arm (inclination velocity)	arm	field	2008–2013	Norway, USA
[13,14,35,46,63,64,65,66,67,68,69,70,71,72,73,74,75,76,77,78,79,80,81,82,83]	accelerometers only	arm (generalized velocity), arm (generalized velocity) and trunk [46,74,75,76,80,82,83]	arm, arm and trunk [46,74,75,76,80,82,83]	field	2002–2018	Sweden, Denmark, Brazil
[39,84,85,86,87,88]	accelerometers only	-	arm, arm and trunk [88]	simulated	2001–2015	Sweden, Brazil, USA
[32,89]	accelerometers only	arm (generalized velocity)	arm	simulated	2013, 2016	Sweden
[90,91,92,93,94]	accelerometers with gyroscopes	-	arm and trunk	field	2014–2020	Sweden, France, Italy, Canada, USA
[44,45,95,96,97]	accelerometers with gyroscopes	arm (inclination velocity), arm and trunk [44,95]	arm, arm and trunk [44,45,95,97]	field	2016–2021	USA
[98,99,100,101,102,103,104]	accelerometers with gyroscopes	-	trunk	field	2007–2018	Germany
[105,106,107,108]	accelerometers with gyroscopes [106,107,108] or magnetometers [105]	-	arm, arm and trunk [106,107,108]	simulated	2009–2017	France, USA
[37,109,110]	accelerometers with gyroscopes	arm (inclination velocity), trunk [110]	arm	simulated	2016–2020	Italy, USA
[40]	accelerometers with gyroscopes	arm (generalized velocity)	arm	simulated	2017	Sweden

The first aim of this study was to investigate the effects of sensor types (using accelerometers only versus using accelerometers with gyroscopes) on measures of postures and movements of the arm and of the trunk in a field study. The second aim was to investigate the effects of angular velocity computational methods, i.e., inclination velocity and generalized velocity, on measures of movements of the arm.

## 2. Materials and Methods

The computations in this study were carried out on data collected in the field, hence there were no standardized experimental movements, and instead real work movements were included.

### 2.1. Participants

This study included half-workday recordings of 38 (13 females and 25 males, 35 right-handed and 3 left-handed) warehouse workers (N = 38) performing their normal work. The demographics of the participants are shown in Table 2. All the participants gave their written informed consent prior participation into the study, which was approved by the Regional Ethics Committee in Stockholm, Sweden (2017/1586-31/4).

### 2.2. Work Tasks

**Figure 1:**
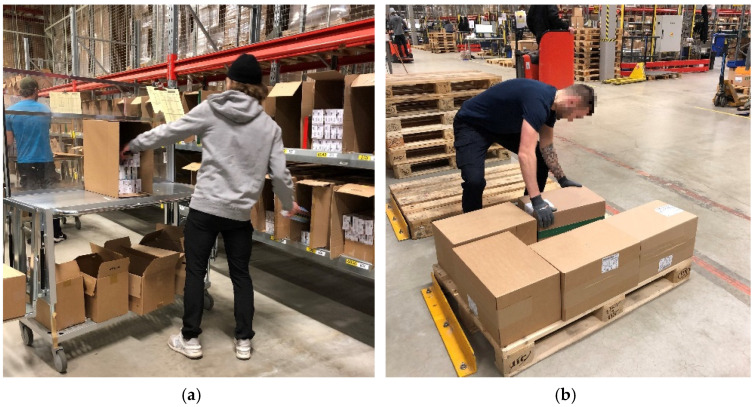
Samples of work tasks for (**a**) order picking and (**b**) palletizing.

The workers performed one of two manual handling tasks: order picking or palletizing (Figure 1). Both tasks involved frequent arm movements, such as grasping, lifting and moving of items with one or two hands.

The order-picking tasks were performed in separate work areas of about 40–50 m^2^ that were enclosed by shelves, where single packages and bundles of packages (0.22–11 kg) containing consumer products were stored from ankle to above shoulder level. The packages were picked following a picking order containing information of item names, quantity, and storage location. The packages were manually picked from the shelves into cardboard boxes (dimensions from 20 × 20 × 15 cm to 45 × 30 × 20 cm) that were placed on hand-pushed carts at about waist level. It took about 5–10 min to complete one order, which included about 50–60 packages. During a normal workday, each order picker completed about 45–50 orders, which corresponded to about 2250–3000 packages. The order pickers worked in a standing position or walking, with no possibility for sitting.

When the orders were completed, the cardboard boxes were manually lifted onto a conveyor belt line, which transferred the boxes to the palletizing stations. At the palletizing station, the cardboard boxes (0.40–12 kg) were manually lifted onto a pallet. The pallet was placed on the floor, about 1 m from the line. There, the cardboard boxes were stacked on top of each other up to a level of 180–190 cm. Each palletizer handled the orders of 3–4 order pickers, which summed up to about 135–200 orders per workday.

### 2.3. Measurements

**Figure 2:**
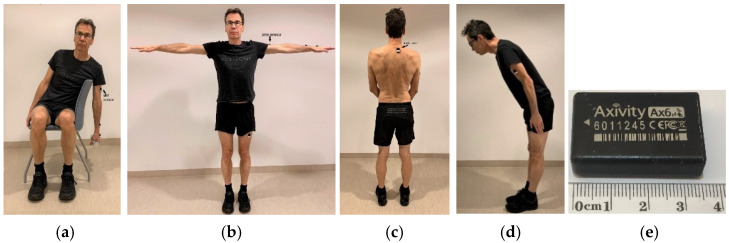
Calibration procedures for the arm and the trunk showing (**a**) the reference posture for the arm, (**b**) the posture for determining the direction of the sensor on the arm, (**c**) the reference posture for the trunk, (**d**) the posture for determining the direction of the sensor on the trunk, and (**e**) the AX6 inertial measurement unit.

The postures and movements of the subjects’ trunk and dominant arm were recorded with two six-axis inertial measurement units (IMUs) (AX6, Axivity Ltd., Newcastle, UK, dimensions: 23 × 32.5 × 8.9 mm, mass 11 g). The AX6 (Figure 2e) builds on the triaxial accelerometer AX3 (Axivity Ltd., Newcastle, UK), which has previously been validated for recordings of physical activity [112,113]—and has previously been used for field measurements of arm postures [53]. Both the AX3 and the AX6 are equipped with a triaxial accelerometer, but the AX6 also has a triaxial gyroscope. For this study, the accelerometer range was set to ±8 g, the gyroscope range to ±1000°/s, and the sampling frequency to 25 Hz. This sampling frequency was chosen since it has been shown that there is very little signal power in work–life movements, in frequency bands above 5 Hz [34], and to avoid unnecessarily large data files. The AX6 has an on-board memory. During the measurements, the data was sampled and stored on the unit. After the measurements, the data was transferred via a USB cable to a computer, where digital filtering and analyses were carried out. The IMU on the dominant arm was positioned with its superior edge just distal to the insertion of the medial deltoid muscle [35], while the IMU on the trunk was placed slightly to the right-hand side of the cervico-thoracic spine at the level of the thoracic vertebrae 1–2 [114]. The IMUs were attached using double-sided adhesive tape (VIP3SC) and fixed with a polyurethane film (Opsite Flexifix) [115]. Four reference postures were performed once by each participant and were used in the calibration procedures for the arm and the trunk (Figure 2a–d). The data for these postures were measured before the collection of actual work postures and movements.

For the arm, the reference position (0° arm elevation) was calculated as the median value of a 3-s window when the subjects were seated, leaning with the trunk laterally over the backrest of the chair, and hanging and relaxing the arm vertically while holding a 2 kg dumbbell in the hand (Figure 2a) [35]. To denote the upward direction of the arm, the subjects were told to abduct both arms 90° and to hold the posture for 10 s (Figure 2b). The median value of a stable 3-s window of this posture was used [35]. For the trunk, the subjects were asked to stand up straight looking forward at eye level, to rise up on their toes and slowly return down to a posture with the full shoe sole on the floor, and then stand still in full balance in an upright position (Figure 2c) [35]. The coordinates of this reference position (0° inclination) were computed as the mean value of a 3-s window. To denote the forward orientation of the trunk, the median value of a 3-s window of a forward bow of the trunk at an arbitrary angle was used (Figure 2d) [35].

### 2.4. Data Processing

**Figure 3:**
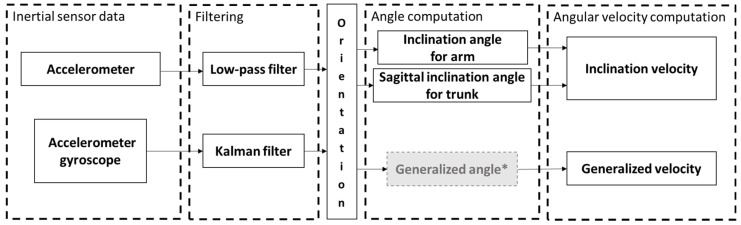
Flowchart of the data processing procedures. * The generalized angles were not included in this study.

Figure 3 shows the data processing steps that were used to calculate the desired angles and angular velocities. Comparisons in this study were conducted between the sensor fusion approaches, i.e., using single data source from accelerometers (*acc*), and using dual data sources from accelerometers and gyroscopes (*acc+gyro*), and/or between the angular velocity computational methods, namely inclination velocity and generalized velocity.

#### 2.4.1. Inertial Sensor Data

The inclination angles and the angular velocities of the arm and the trunk were defined in the coordinates of the corresponding body parts. As the IMUs were configured with an intrinsic coordinate system, readings from the IMUs were first transformed to the body part coordinates, as follows:

(1) $$\vec{vector_{body}}=R\cdot \vec{vector_{sensor}}$$

where *R* is defined by the rotation angles needed for this transformation:

(2) $$R=R_{z}\left(\theta_{z}\right)\cdot R_{y}\left(\theta_{y}\right)\cdot R_{x}\left(\theta_{x}\right)$$

After the transformation, the outcomes were used as an input for the further steps.

#### 2.4.2. Filtering

The postures of the arm and trunk were derived from the orientation of the sensor in relation to the accelerometer signal, caused by gravity, that were acquired from the IMUs. The orientations were calculated by two approaches: (1) using only accelerometers without sensor fusion (*acc*), and (2) using a Kalman filter algorithm with bias compensation to integrate gyroscopes with accelerometers (*acc+gyro*) [109].

For the *acc* approach, the readings from the accelerometers were filtered by a 5-Hz low-pass Blackman window-based filter [34]. For the *acc+gyro* approach, a complementary or a Kalman filter is normally chosen. In this study, we chose, in accordance with recommendations by Chen et al. [109], a Kalman filter with the coefficients used by Chen et al. [109], namely (at a sampling frequency of 128 Hz as used by Chen et al., which we obtained after resampling), 0.005 rad/s for the gyroscope white noise, 0.0005 rad/s^2^ for the gyroscope bias noise and 0.1 m/s^2^ for the accelerometer white noise [109]. After the Kalman filter, the angle signal was again resampled, to the original frequency of 25 Hz.

The results of this step were orientations of the corresponding body part, which were further used to calculate angles and angular velocities (Figure 3).

#### 2.4.3. Angle Computation

The calculation of the arm inclination angle was adapted from Yang et al. [40], and was based on the angular displacement from the reference position (Figure 2a):

(3) $$inclination\;angle_{i}=2\arcsin\left(\frac{\left|\vec{g_{i}}-\vec{g_{ref}}\right|}{2}\right)\in \left[0{}^{\circ},\;180{}^{\circ}\right]$$

where $\vec{g_{i}}$ is the gravitation vector at any given time i as $\left(\begin{array}{c} \begin{array}{c} x_{i}\\ y_{i} \end{array}\\ z_{i} \end{array}\right)$ and $\vec{g_{ref}}$ is the gravitation vector at the reference position as $\left(\begin{array}{c} \begin{array}{c} x_{ref}\\ y_{ref} \end{array}\\ z_{ref} \end{array}\right)$. Both vectors are normalized to the unit sphere.

The sagittal inclination angle of the trunk was calculated as the forward/backward projections in Hansson et al. [34].

#### 2.4.4. Angular Velocity Computation

For the arm, both the inclination velocity and generalized velocity were calculated. The inclination velocity of each body part was determined as the derivative of the inclination angle with respect to time.

To derive the generalized velocity, the generalized angle was first calculated by using an equation equivalent to the one used by Hansson et al. [34]:

(4) $$generalized\;angle_{i}=2\arcsin\left(\frac{\left|\vec{g_{i}}-\vec{g_{i-1}}\right|}{2}\right)\in \left[0{}^{\circ},\;180{}^{\circ}\right]$$

where $\vec{g_{i}}$ is the gravitation vector at any given time i as $\left(\begin{array}{c} \begin{array}{c} x_{i}\\ y_{i} \end{array}\\ z_{i} \end{array}\right)$, and $\vec{g_{i-1}}$ is the gravitation vector of the previous sample as $\left(\begin{array}{c} \begin{array}{c} x_{i-1}\\ y_{i-1} \end{array}\\ z_{i-1} \end{array}\right)$. Both vectors were normalized to the unit sphere. The generalized velocity was, then, calculated as the derivative of the generalized angle with respect to time. For the trunk, only the sagittal inclination velocity was calculated. It was calculated as the derivative of the sagittal inclination angle. All velocity values were finally converted to their absolute value.

#### 2.4.5. Statistical Analysis

Postural angles and angular velocities were first computed as individual measures including mean values, percentiles, percentile ranges, and proportions of time meeting specific criteria. Those individual measures were then averaged and presented on group level as group measures. The effects of the sensor types and the angular velocity computational methods were evaluated by comparing the group measures that were obtained by the corresponding methods, such as using accelerometers only *(acc)* versus using accelerometers with gyroscopes (*acc+gyro*). The Shapiro–Wilk test was used for examining normality of data. Additionally, since t-test is robust to slightly skewed and kurtotic distributions, distributions having a skewness below 2.0 and kurtosis below 6.0 were considered (sufficiently) normally distributed as well [116]. For sufficiently normally distributed data, paired t-test were applied, while the Wilcoxon signed-rank test was used for non-normally distributed data. A *p*-value of 0.01 was used to denote statistically significance due to the multiple comparisons among the measures. Additionally, a correlation analysis was performed to compare the angles and the velocities (10th, 50th and 90th percentile) between *acc* and *acc+gyro*. The Pearson correlation coefficient was calculated after the confirmation of the normality of the data. A linear model was used to fit the inclination data, and a zero-intercept linear model was used for the angular velocity data. The distribution of the differences over the average values of each compared pair was further plotted in a Bland–Altman plot. The statistical analysis was performed in MATLAB R2019b (MathWorks, Inc., Natick, MA, USA).

## 3. Results

**Figure 4:**
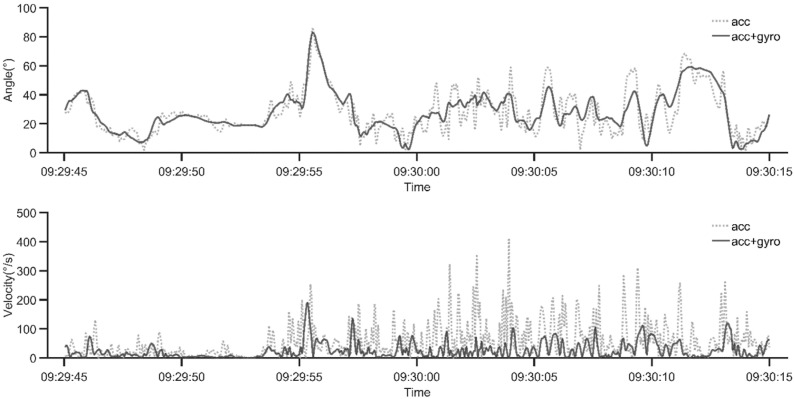
A sample illustrating the differences of the inclination angle (**above**) and the inclination velocity (**below**) of the arm, using measurements based on only accelerometers (*acc*), and using accelerometers with gyroscopes (*acc*+*gyro*).

Both the inclination angles and the angular velocities differed between the sensor types; that is, accelerometers alone (*acc*) and accelerometers with gyroscopes (*acc+gyro*) (Figure 4).

### 3.1. Comparison of Inclination Angles

For the arm, Table 3 shows the group mean values and standard deviations (SD) of all measures. The mean value and the 1st–90th percentiles of the inclination angle measured by *acc* were significantly lower (*p* < 0.0001) than the corresponding values by *acc+gyro*. However, all differences were less than 2°. There was no significant difference in the 99th percentile arm inclination angle or in the percentile range between *acc* and *acc*+*gyro*. As for the distribution of the inclination angles in time, the proportion of time of arm inclination <20° was significantly higher for *acc* (*p* < 0.0001) than by *acc*+*gyro*, while the proportion of time of the arm inclination angles >30°, >45°, >60° (*p* < 0.0001) and >90° (*p* < 0.001) were lower by *acc* than by *acc*+*gyro*. Again, the differences were small (all < 3%).

For the trunk sagittal inclination (Table 3), no significant differences were observed in either the mean or median trunk inclination angles between *acc* and *acc*+*gyro*, while the 1st–25th percentiles of the trunk sagittal inclination angles (forward bending) were significantly lower for *acc* than for *acc*+*gyro* (*p* < 0.0001), and the 75th–99th percentile angles were significantly higher (*p* < 0.0001) for *acc* than for *acc*+*gyro*. These differences were all less than 2°. The percentile range was 2.7° higher for *acc* than for *acc*+*gyro* (*p* < 0.001). The proportion of time of trunk inclination <20° was significantly lower for *acc* than for *acc*+*gyro*, and the proportion of time of trunk inclination angles >30°, >45° and >60° were higher for *acc* than for *acc*+*gyro* (*p* < 0.001). Again, the differences were all small (<1.5%).

**Figure 5:**
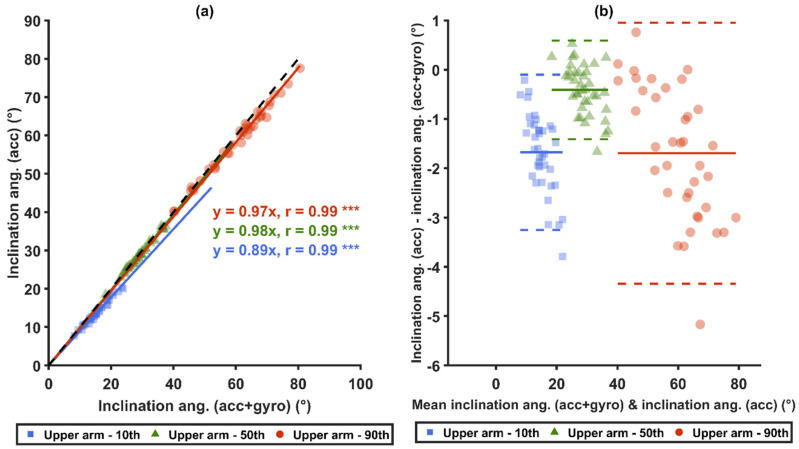

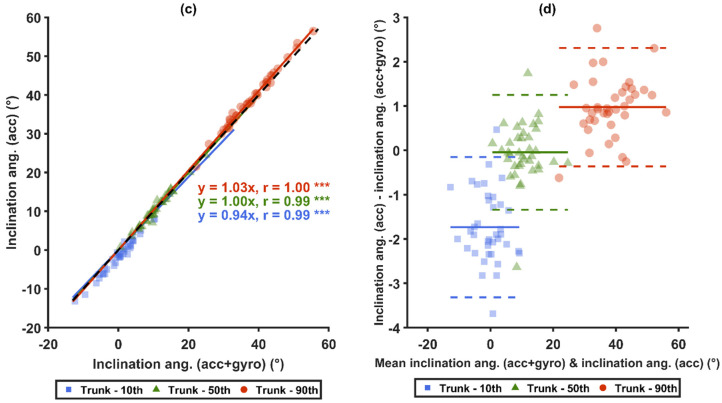
Comparisons of inclination angles measured between the two sensor types (*acc* versus *acc*+*gyro*) for the arm and for the trunk. Graphs (**a**,**b**) show the arm inclination angles of *acc* and *acc*+*gyro*, while (**c**,**d**) show the trunk sagittal inclination angles between *acc* and *acc*+*gyro*. In (**a**,**c**), the black-dashed lines represent unity, and the colored-solid lines represent the zero-intercept linear prediction models of the corresponding percentiles. In (**b**,**d**), Bland–Altman plots are shown: the colored-solid lines mark the mean values of the differences of the corresponding percentiles, and the dashed lines represent ±1.96 SD. *** denotes *p* < 0.0001.

As shown in Figure 5a,c, the two sensor types (*acc* and *acc+gyro*) were strongly correlated (r > 0.98) for the 10th, 50th and 90th percentiles of both the arm inclination angles and the trunk sagittal inclination angles. As illustrated by the Bland–Altman plots in Figure 5b,d, the average differences in inclination angles between *acc* and *acc*+*gyro* were the smallest at the 50th percentile for both the arm and the trunk. For the arm, these average differences also showed a tendency to increase with increased percentile angles (Figure 5b). For the trunk, this tendency was not prominent (Figure 5d).

### 3.2. Comparisons of Angular Velocities

Statistically significant differences were found among all the measures from both arm angular velocity computational methods and from both sensor types in all three comparison pairs: (1) *acc* versus *acc+gyro*, (2) generalized velocity versus inclination velocity, and (3) generalized velocity with *acc* versus inclination velocity with *acc+gyro* (Table 4).

When comparing the two sensor types (Table 4), the mean values and the 5th–99th percentiles of both angular velocities from *acc* were between 60% and 207% higher than the corresponding measures from *acc+gyro* (*p* < 0.0001). The differences increased with increased velocity. For both angular velocity computational methods, the proportion of time at slow movements (<5°/s) in all angles and in neutral arm inclination angles (<15° and <20°) were significantly lower from *acc+gyro* than from *acc* (*p* < 0.0001), and the proportion of time at fast motion (>90°/s) from *acc* was significantly higher than from *acc+gyro* (*p* < 0.0001).

When comparing the two angular velocity types (Table 4), the mean values and the 5th–99th percentiles of the generalized velocity were between 26% and 267% higher than those of the inclination velocity for both sensor types (*p* < 0.0001). The differences increased with the increase of the angular velocity. Within both sensor types, the proportion of time at slow movements (<5°/s) in all angles and in neutral arm inclination angles (<15° and <20°) were all significantly lower for the generalized velocity than for the inclination velocity (*p* < 0.0001). The proportion of time at fast movements (>90°/s) of the generalized velocity was significantly higher than of the inclination velocity (*p* < 0.0001).

When comparing the generalized velocity from *acc*, which was used in recommendations from Balogh et al. [14] and Arvidsson et al. [48], to the inclination velocity from *acc+gyro*, the mean values and all the 5th–99th percentiles of generalized velocity from *acc* were between 167% and 780% higher than the latter (*p* < 0.0001). The differences were from 3.9°/s for the 5th percentile up to 259.9°/s for the 99th percentile. The proportion of time at slow movements (<5°/s) in all angles and in neutral arm inclination angles (<15° and <20°) for the generalized velocity from *acc* were significantly lower than for the inclination velocity from *acc+gyro* (*p* < 0.0001), and the proportion of time at fast motion (>90°/s) for the generalized velocity from *acc* was significantly higher than ditto from *acc+gyro* (*p* < 0.0001).

**Figure 6:**
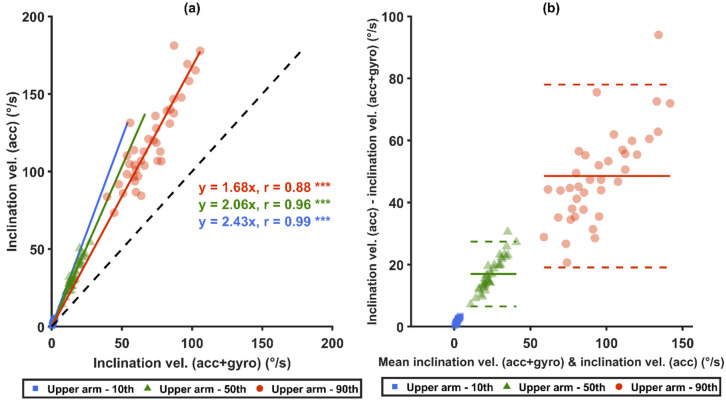

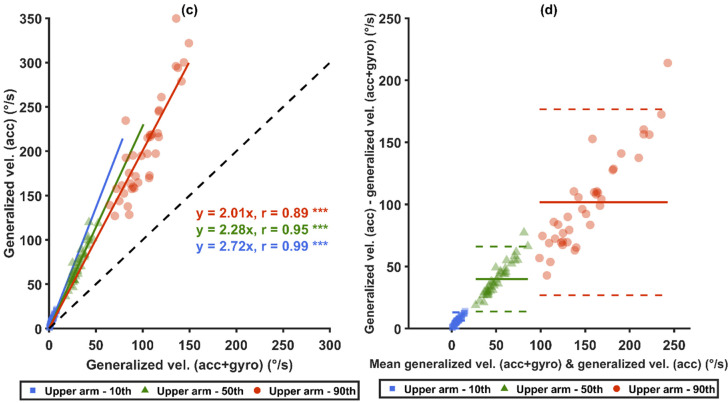
Comparisons between arm angular velocities measured from different sensor types. Graphs (**a**,**b**) show the inclination velocities of *acc* and *acc*+*gyro*, while (**c**,**d**) show the generalized velocities of *acc* and *acc*+*gyro*. Correlation plots are shown in (**a**,**c**): the black-dashed lines represent unity, and the colored-solid lines represent the zero-intercept linear prediction models of the corresponding percentiles. In (**b**,**d**), Bland–Altman plots are shown: the colored-solid lines mark the mean value of the differences, and the dashed lines represent ±1.96 SD. *** denotes *p* < 0.0001.

**Figure 7:**
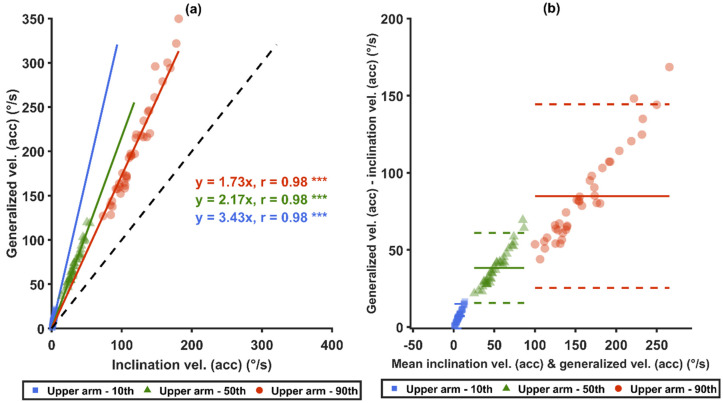

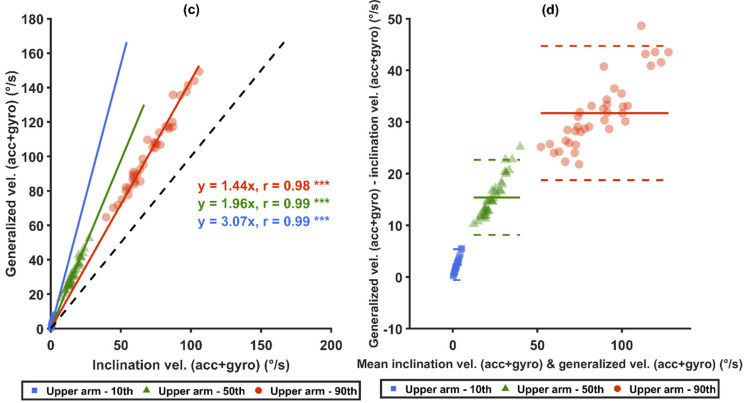
Comparisons between the different arm angular velocity computational methods. Graphs (**a**,**b**) present the generalized velocities and the inclination velocities from *acc*, while (**c**,**d**) present the generalized velocities and the inclination velocities from *acc*+*gyro*. Correlation plots are shown in (**a**,**c**): the black-dashed lines represent unity, and the colored-solid lines represent the zero-intercept linear prediction models of the corresponding percentiles. In (**b**,**d**), Bland–Altman plots are shown: the colored-solid lines mark the mean value of the differences, and the dashed lines represent ±1.96 SD. *** denotes *p* < 0.0001.

**Figure 8:**
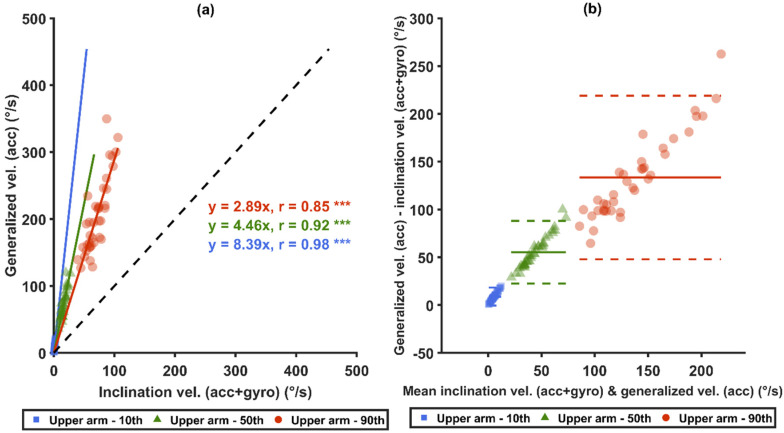
Comparisons between the generalized velocities from *acc* and the inclination velocities from *acc*+*gyro*. Correlation plots are shown in (**a**): the black-dashed lines represent unity, and the colored-solid lines represent the zero-intercept linear prediction models of the corresponding percentiles. In (**b**), Bland–Altman plots are shown: the colored-solid lines mark the mean value of the differences, and the dashed lines represent ±1.96 SD. *** denotes *p* < 0.0001.

Strong correlations (r ≥ 0.85) were found for the 10th, 50th and 90th percentiles between the two sensor types, i.e., *acc* and *acc*+*gyro* (Figure 6a,c), and between the two angular velocity computational methods (Figure 7a,c). Significant correlations were also found between the generalized velocity for *acc* and the inclination velocity for *acc+gyro* (Figure 8a). As illustrated by the Bland–Altman plots in Figure 6b,d, Figure 7b,d and Figure 8b, the differences in the three mentioned comparisons showed a tendency to increase with increased velocities.

When comparing the two sensor types for the trunk (Table 5), the mean values, the 5th–99th percentiles and the percentile range of the sagittal inclination velocities from *acc* were between 117% and 200% (relative differences calculated from Table 5) higher than from *acc+gyro* (*p* < 0.0001). The proportion of time at slow movements (<5°/s) in all angles and in neutral trunk inclination angles (between −10° and 20°, <15°, and <20°) from *acc* were all less than half than those from *acc+gyro* (*p* < 0.0001), and the proportion of time at fast motion (>90°/s) from *acc* was about 17 times higher than from *acc+gyro* (*p* < 0.0001).

**Figure 9:**
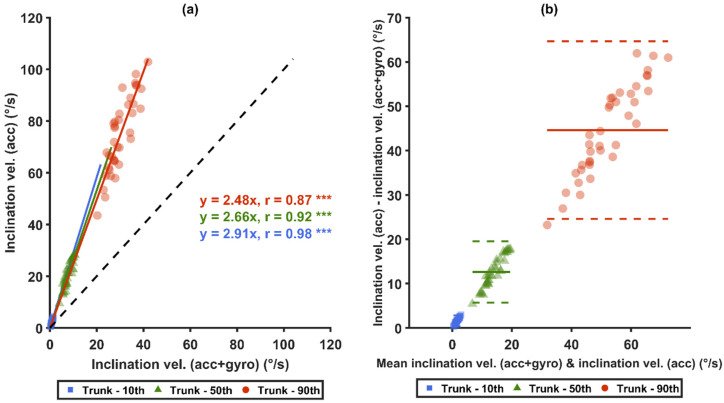
Comparisons of trunk sagittal inclination velocities measured between two sensor types (*acc* and *acc*+*gyro*): (**a**) shows the correlation plot: the black-dashed lines represent unity, and the colored-solid lines are the zero-intercept linear prediction model of the corresponding percentile; (**b**) shows the Bland–Altman plot: the colored-solid lines mark the mean value of the differences, and the dashed lines represent ±1.96SD. *** denotes *p* < 0.0001.

As illustrated in Figure 9a, the two sensor types (*acc* and *acc+gyro*) were correlated (r > 0.86) for the 10th, 50th, 90th percentiles of the sagittal inclination velocities. The Bland–Altman plots (Figure 9b) show that differences between the sensor types increased with the increase of the measured values.

## 4. Discussion

This field study included half-workday measurements of 38 warehouse workers. When compared to using accelerometers with gyroscopes—using accelerometers alone had a significant but generally small effect (<2°) on the measures of inclination angles. However, the effects of sensor types on the measures of both angular velocity computational methods were significant and substantial. Angular velocities from *acc* were about twice as high as those from *acc+gyro* for the two angular velocity computational methods. Furthermore, the arm generalized velocity, which included arm axial rotation, showed significantly higher values (26–267%) in measures of arm movements than those of the inclination velocity.

### 4.1. Methodological Considerations

One strength of this study was that it comprised measurements of trained warehouse workers performing their normal occupational work tasks, as opposed to the simulated work tasks or movements as those used in previous studies that compared the effects of sensor types on measurements of angles and velocities. In comparison to simulated work, real occupational work likely includes a broader range of complexity of movements. As shown by Yang et al. [40], the increased complexity in tasks (simulated/real work versus simple arm swing) may amplify the errors introduced by sensor types and angular velocity computational methods even when the velocity range is similar (see Section 4.2 and Section 4.3). However, future studies are required to further investigate the extent to which the differences observed in this study also exist at a similar level in a broad range of occupations.

Another strength of this study is the sample population: both male and female workers were included. Although a balanced gender-sample could have been argued for, the female proportion in this study (34%), is relatively high when compared to the 22% among warehouse (and terminal) workers in Sweden [117]. The sample size (N = 38) was substantially larger than those in the previous laboratory-based studies that included simulated work; that is, 10 participants [40] and 11 participants [109]. As always in workplace exposure assessments, there were variances between workers (see Table 3, Table 4 and Table 5). The studied work was of repetitive nature, so per worker the half workday data collection should be sufficient. Considering the very low *p*-values in the comparisons, the sample size was likely to be sufficiently high for the sensor and computational comparisons that were performed. Other methodological procedures may also contribute to differences in results between studies, such as the reference positions [32,68], the placement of the sensors [85,118], and possibly soft-tissue artifacts [119,120]. In the current study, such variances were overcome by the use of paired comparisons.

Optical systems with multiple cameras are usually considered as the gold standard for motion tracking. However, this study targeted to evaluate the difference between commonly used sensor types and angular computational methods. Therefore, an optical motion tracking system was not suitable given the aims of this study. Additionally, several studies have demonstrated that inclination velocities from IMUs that applies a fusion of accelerometer and gyroscopes are close to those of optical systems [37,40,91,109]. Third, since the study was carried out in a real occupational setting of ambulatory work, the feasibility of using optical systems is restricted.

### 4.2. The Effects of Sensor Types: acc Versus acc+gyro

For postures, the result of *acc* showed similar percentile values of arm inclination angles (<2°) as those based on the accelerometers with gyroscopes, *acc+gyro*, and the differences of the angles in percentiles between the two sensor types did not exhibit a dependency on the measured value. This result is consistent with that of Chen et al. [37], who reported differences <1.8° in all percentiles between *acc* and *acc+gyro* during movements of slow arm swings.

In contrast, there were large relative differences in arm velocity between the two sensor types. For example, the mean inclination velocities from *acc* were 2–3 times higher than those from *acc*+*gyro*, i.e., 1.8°/s higher for the 10th percentile, 16.9°/s higher for the 50th percentile and 48.5°/s higher for the 90th percentile. These differences were larger when compared to those of both Yang et al. [40] and Chen et al. [37]. Yang et al. [40] reported differences in inclination velocity attributed to the sensor type of <4°/s at the 50th percentile and <12°/s at the 90th percentile during arm swings for inclination velocities that were similar to those in this study. Chen et al. [37] reported differences in inclination velocity attributed to the sensor type of <30°/s at the 90th percentile during arm swings of inclination velocities that were similar or faster than those in this study. One reason for these differences could be that Chen et al. [37] used a 3-Hz low-pass filter for *acc* while in this study a 5-Hz low-pass filter [34] was used. Another reason, although probably less important, could be hardware differences given that the parameters for the Kalman filter were the same as Chen et al. [109] (after resampling to the same frequency), but the accelerometers and gyroscopes in this study were not identical to those in Chen et al. [37], Chen et al. [109]. A third reason could be the higher complexity of movements in the real work. As explained by Bernmark and Wiktorin [39], centripetal acceleration is a major source of errors for accelerometer-based measurement. The more complex the work task becomes, the more and higher centripetal acceleration is involved; hence, the larger the errors become.

The differences in the angular velocities between the sensor types increase from lower percentile rank to the higher percentile rank, which was also observed by both Yang et al. [40] and Chen et al. [37]. This may be explained by two reasons: velocity and percentile rank. For velocity, it was demonstrated by Bernmark and Wiktorin [39] that centripetal acceleration is a major source of errors for accelerometers, and it is proportional to the square of angular velocity. The higher the percentile rank is, the higher the velocity value is; ergo, the higher the centripetal acceleration becomes, which leads to higher differences between the two sensor types. Another result in this study that likely resonates this explanation is that although a strong correlation (r > 0.85) was found between the two sensor types within the 10th, 50th and 90th percentiles for both inclination angles and angular velocities, the differences were also non-linearly dependent on the velocities. Only one brand of sensors was used in this study. However, systematic difference in angular velocities between accelerometers only and accelerometers fused with gyroscopes are in agreement with previous laboratory-based studies where other brands of sensors have been used [37,40]. Current sensors on the market are in general of low noise and high precision. Therefore, the large differences observed in the current study is unlikely due to the choice of brands of sensors, but due to the errors from using only accelerometers.

In summary, this study clearly shows that velocities from studies that have used different sensor types should not be compared directly, but may be compared after a conversion. In this study, the *acc* velocities were found to be approximately twice as high as the *acc+gyro* velocities (Figure 6a,c). However, further studies are needed to establish a more detailed conversion model.

### 4.3. The Type of Angular Velocity: Generalized Velocity versus Inclination Velocity

Our results show that the two angular velocity computational methods resulted in substantially different values. When using the same sensor type, the 50th and the 90th percentiles of the generalized velocity for *acc* were 116.4% and 71.3% higher than those of inclination velocity, respectively. The corresponding comparison for *acc+gyro* was 97% and 45%, respectively. These substantial velocity differences are consistent to the findings in Yang et al. [40].

The velocity differences between two sensor types were larger for the generalized velocities than for the inclination velocities, which agrees with the results by Yang et al. [40]. It is suggested that the generalized velocity is more sensitive to errors introduced by the sensor type. The generalized velocity consists of the inclination velocity and the velocity of the axial rotation, and it must be equal to or larger than the inclination velocity. This addition of a velocity component introduces an additional component of centripetal acceleration, which likely explains why generalized velocity has a higher sensitivity to measurement errors than inclination velocity does.

### 4.4. Velocity Conversions

As shown in Figure 8a, the median *acc+gyro* inclination velocity can, in this study with the present median velocities, approximately be converted to median *acc* generalized velocity by a conversion factor of 4.46. Alternatively, one can first convert the median *acc+gyro* inclination velocity to the median *acc* inclination velocity by a factor of 2.06 (Figure 6a), and then convert that result to the median *acc* generalized velocity by a factor of 2.17 (Figure 7a). This gives a total factor of 4.47, which is close to the measured factor of 4.46 for the one-step conversion in Figure 8a.

### 4.5. Practical Implications

Laboratory studies on this issue have been performed previously, but, to the best of our knowledge, this is the first study that has evaluated the effects of sensor types on assessments of postural loads and movements, and the effects of angular velocity computational methods, in a real occupational work.

For postures, there were only minor differences between the percentiles from different sensor types, which indicates that *acc* and *acc+gyro* studies can be directly compared. Meanwhile, for velocities, the sensor types and the angular velocity computational methods have substantial effects on the values: the average median *acc* inclination velocity was about twice as high as that from *acc+gyro*, and the median *acc+gyro* generalized velocity was about twice as high as that of inclination velocity. When combining the two factors, the median generalized velocity from *acc* was about 4.5 times as high as the median inclination velocity from *acc+gyro*. Thus, angular velocities measured with different methods should not be compared without a conversion. In some of the arm velocity studies in Table 1 [45,96,97], results of inclination velocity from *acc+gyro* have been compared with studies using the generalized velocity from *acc*. Hence, in the future, such comparisons should be done after conversions, either by using the factors above or using more detailed models.

Additionally, action levels and threshold limit values for wrist and arm velocities have been recommended [14,48]. The recommended threshold limit value for arm velocity is 60°/s and is based on generalized velocity and *acc* measurements in many different occupational groups [14,48]. If the results of *acc+gyro* and/or inclination velocity should be compared with that threshold limit value, then the level needs to be lowered in accordance with the previous paragraph. By matching the individual distributions of the other velocities to the *acc* generalized velocities, more accurate thresholds could be acquired for each of the other velocities—for instance, if an *acc* generalized velocity of 60°/s was at one subject’s 56th percentile, the equivalent thresholds for the other three velocities can for this subject be found at the 56th percentile of the corresponding velocity. According to this transformation, using the average of all subjects, the threshold level of 60°/s should be lowered to 26.6°/s for *acc* inclination velocity, to 26.4°/s for *acc+gyro* generalized velocity, and to 12.9°/s for *acc+gyro* inclination velocity. The results showing that conversion is needed have practical implications for both researchers and, e.g., occupational health services aiming to use the recommended threshold limit values or comparing results to those obtained using different sensor types or angular velocity computational methods.

As shown in Figure 6, Figure 7 and Figure 8, the factors mentioned above (i.e., 2 and 4.5) are approximate, and the relative differences increase with the velocity. As the current study includes only one occupational group, the converted recommended threshold limit values may not be generalized to other occupations. Therefore, further studies are required to explore optimal nonlinear conversion models, and to determine if specific conversion models are needed in different occupations.

## 5. Conclusions

Previously, inclination and velocity results of different sensors and of different computational methods have been compared as if they were fully comparable. In the present results both the sensor types and the angular velocity computational methods showed significant effects on the measures of postures and movements in warehouse work. For the posture angle percentiles, the differences were below clinical relevance (<2°), which indicates that the 10th, 50th and 90th percentiles from accelerometers (*acc*) can be compared with percentiles from accelerometers with gyroscopes (*acc+gyro*). For movements, the differences of the velocity percentiles were substantial, for instance, at the 50th percentile: *acc* was about double as high as *acc+gyro*, e.g., 33°/s versus 16°/s for inclination velocity, and for the angular velocity computational methods, the generalized velocity was about double as high as the inclination velocity, e.g., 31°/s versus 16°/s for *acc+gyro*. The generalized velocities from *acc* were about 4.5 times as high as the inclination velocities from *acc+gyro*, e.g., 71°/s versus 16°/s at the 50th percentile. Hence, movement velocities of different computational methods and from different sensor types should not be directly compared. Both researchers and practitioners, who aim to compare velocities with recommended threshold limits, should check if they can compare directly or if a conversion is needed.

This study included only one occupational group. Future studies are needed to investigate the effects of sensor types and angular computational methods on kinematic measurements among other occupational groups. There is a need for conversion models between data obtained by different sensor types and angular velocity computational methods.

## Figures and Tables

**Table 2:** The demographics of the 38 participants. In self-rated work ability, “0” indicates not being able to work and “10” corresponds to having a work ability at its best [111].

Characteristics	Statistics
Male, count (%)	25 (66%)
Age, mean (standard deviation, SD)	25 (8) years
Body mass, mean (SD)	76 (11) kg
Statue, mean (SD)	178 (8) cm
Work experience, (%)	
<1 year	29%
1–2 years	20%
3–5 years	43%
>10 years	9%
Self-rated work ability, mean (SD)	8.6 (1.4)

**Table 3:** Group mean values and standard deviations (SD) of measures for the arm inclination angles and the trunk sagittal inclination angles measured using only accelerometers (*acc*) and by using accelerometers with gyroscopes (*acc*+*gyro*), and the group mean differences (SD) of the comparison between those two sensor types (N = 38).

	*acc*		*acc+gyro*		*acc - acc+gyro*		
	Mean (SD)		Mean (SD)		Mean (SD)		
**Upper arm**							
** Inclination angle**							
Mean (°)	33.3	(4.7)	34.3	(5.0)	−0.9	(0.5)	***
**Percentile (°)**							
1st	5.3	(1.5)	6.8	(2.1)	−1.5	(0.8)	***
5th	10.6	(2.6)	12.5	(3.4)	−1.9	(0.9)	***
10th	14.0	(3.1)	15.6	(3.7)	−1.7	(0.8)	***
25th	20.3	(3.7)	21.1	(4.0)	−0.9	(0.5)	***
50th	28.8	(4.2)	29.2	(4.5)	−0.4	(0.5)	***
75th	41.6	(5.8)	42.4	(6.4)	−0.8	(0.9)	***
90th	58.9	(9.1)	60.6	(10.0)	−1.7	(1.4)	***
99th	100.2	(13.8)	100.1	(13.6)	0.1	(1.0)	
**Percentile range (°)**							
10th–90th	44.9	(7.8)	44.9	(8.7)	0.0	(1.5)	
**Proportion of time (%)**							
<20°	25.6	(9.9)	23.0	(11.2)	2.6	(1.7)	***
>30°	47.0	(11.1)	48.2	(12.3)	−1.2	(1.5)	***
>45°	20.9	(7.2)	21.9	(7.6)	−1.0	(0.9)	***
>60°	9.9	(4.4)	10.8	(4.9)	−0.9	(0.7)	***
>90°	2.2	(1.4)	2.3	(1.6)	−0.1	(0.2)	**
**Trunk**							
** Inclination angle in the sagittal plane**							
Mean (°)	15.0	(4.8)	15.2	(4.7)	−0.2	(0.6)	^W^
**Percentile (°)**							
1st	−13.6	(7.7)	−12.0	(8.2)	−1.7	(1.2)	***
5th	−5.1	(5.3)	−3.4	(5.6)	−1.8	(1.0)	***
10th	−1.4	(4.7)	0.3	(4.8)	−1.7	(0.8)	***
25th	4.1	(4.2)	5.1	(4.2)	−1.0	(0.6)	*** ^W^
50th	10.6	(4.6)	10.6	(4.6)	0.0	(0.7)	^W^
75th	22.1	(6.6)	21.6	(6.5)	0.5	(0.6)	***
90th	38.9	(7.4)	38.0	(7.2)	1.0	(0.7)	***
99th	71.3	(5.9)	69.8	(6.3)	1.4	(1.2)	*** ^W^
**Percentile range (°)**							
10th–90th	40.4	(6.2)	37.6	(6.0)	2.7	(1.0)	***
**Proportion of time (%)**							
angle (−10° to 20°)	68.9	(8.6)	70.2	(8.7)	−1.4	(1.2)	***
<20°	71.7	(9.5)	72.4	(9.5)	−0.7	(0.9)	***
>30°	17.2	(7.0)	16.7	(7.0)	0.5	(0.5)	***
>45°	7.7	(3.6)	7.3	(3.5)	0.4	(0.4)	***
>60°	3.2	(1.7)	2.9	(1.6)	0.3	(0.2)	*** ^W^
>90°	-	(-)	-	(-)	-	(-)	

**: *p* < 0.001, ***: *p* < 0.0001, ^W^: the Wilcoxon sign-rank test was used.

**Table 4:** Group mean values and standard deviations (SD) of inclination velocity and the generalized velocity of the arm measured by accelerometers only (*acc*) and accelerometers with gyroscopes (*acc*+*gyro*), and the group mean differences (SD) of the comparisons between the two angular velocity computational methods and the two sensor types (N = 38).

Angular Velocity Computational Method	Incl. Vel. ^a^				Gen. Vel. ^b^				Incl. Vel.			Gen. Vel.			Gen. Vel.-Incl. Vel.						(Incl. Vel. with *acc+gyro*) - (Incl. Vel. with *acc+gyro*)		
Sensor Type	*Acc*		*acc+gyro*		*acc*		*acc+gyro*		*acc-acc+gyro*						*acc*			*acc+gyro*					
	Mean (SD)		Mean (SD)		Mean (SD)		Mean (SD)		Mean (SD)			Mean (SD)			Mean (SD)			Mean (SD)			Mean (SD)		
**Upper arm**																							
** Angular velocity**																							
Mean (°/s)	50.0	(12.3)	27.7	(6.6)	94.2	(26.8)	44.2	(10.1)	22.3	(6.8)	***	50.0	(17.9)	***	44.2	(14.9)	***	16.4	(3.7)	***	66.4	(21.2)	***
**Percentile (°/s)**																							
5th	1.2	(0.6)	0.5	(0.3)	4.3	(2.5)	1.4	(1.0)	0.7	(0.3)	***	2.9	(1.6)	***	3.2	(2.0)	***	0.9	(0.7)	***	3.9	(2.3)	***
10th	3.0	(1.4)	1.2	(0.7)	10.1	(5.4)	3.6	(2.2)	1.8	(0.8)	***	6.5	(3.3)	***	7.1	(4.1)	***	2.4	(1.5)	***	8.9	(4.8)	***
25th	11.5	(4.3)	5.0	(2.0)	31.9	(12.1)	12.8	(4.7)	6.5	(2.4)	***	19.0	(7.6)	***	20.3	(7.9)	***	7.8	(2.8)	***	26.8	(10.3)	***
50th	32.9	(9.3)	15.9	(4.3)	71.2	(20.6)	31.3	(7.9)	16.9	(5.3)	***	39.9	(13.4)	***	38.3	(11.6)	***	15.4	(3.7)	***	55.3	(16.7)	***
75th	69.9	(17.1)	37.7	(9.2)	128.7	(35.3)	61.7	(14.0)	32.2	(9.3)	***	67.0	(23.0)	***	58.8	(18.8)	***	24.0	(5.3)	***	91.0	(27.5)	***
90th	119.0	(27.5)	70.5	(16.6)	203.9	(56.8)	102.2	(22.3)	48.5	(15.0)	***	101.7	(38.2)	***	84.9	(30.4)	***	31.7	(6.6)	***	133.4	(43.6)	***
99th	248.7	(55.8)	155.8	(32.3)	415.7	(118.4)	195.5	(37.8)	93.0	(32.3)	***	220.2	(86.8)	***	167.0	(65.6)	***	39.8	(9.0)	***	259.9	(93.6)	***
**Proportion of time (%)**																							
<5°/s	15.5	(5.4)	25.9	(6.2)	7.4	(4.8)	13.8	(5.5)	−10.4	(2.0)	***	−6.4	(1.8)	***	−8.1	(1.4)	***	−12.1	(1.8)	***	−18.5	(3.2)	***
>90°/s	17.1	(6.6)	6.2	(3.3)	38.8	(11.2)	13.3	(5.9)	10.9	(4.0)	***	25.6	(6.2)	***	21.7	(4.8)	***	7.1	(2.8)	***	32.6	(8.5)	***
**Combined parameter**																							
<15° and <5°/s	1.7	(1.4)	2.8	(2.3)	0.6	(0.9)	1.3	(1.5)	−1.1	(1.1)	*** ^W^	−0.7	(0.7)	*** ^W^	−1.0	(0.7)	***	−1.5	(1.1)	***^W^	−2.2	(1.7)	*** ^W^
<20° and <5°/s	3.4	(2.1)	6.1	(3.5)	1.3	(1.4)	2.9	(2.2)	−2.7	(1.7)	***	−1.6	(1.0)	***	−2.0	(1.0)	***	−3.2	(1.8)	***	−4.8	(2.7)	***

^a^: inclination velocity, ^b^: generalized velocity, ^W^: the Wilcoxon sign-rank test was used. ***: *p* < 0.0001

**Table 5:** The group means (SD) for the trunk sagittal inclination velocity measured by using only accelerometers (*acc*) and by using accelerometers with gyroscopes (*acc*+*gyro*), and the group mean differences (SD) between those two sensor types (N = 38).

	*acc*		*acc+gyro*		*acc-acc+gyro*		
	Mean (SD)		Mean (SD)		Mean (SD)		
**Trunk**							
** Sagittal inclination velocity**							
Mean (°/s)	31.3	(6.5)	12.5	(2.2)	18.8	(4.6)	***
**Percentile (°/s)**							
5th	1.1	(0.5)	0.4	(0.2)	0.7	(0.3)	***
10th	2.4	(1.0)	0.8	(0.3)	1.6	(0.6)	***
25th	7.5	(2.5)	2.7	(0.8)	4.9	(1.7)	***
50th	20.2	(5.0)	7.6	(1.7)	12.6	(3.5)	***
75th	43.3	(9.3)	16.7	(3.1)	26.6	(6.6)	***
90th	74.7	(14.5)	30.1	(5.3)	44.6	(10.2)	***
99th	157.5	(26.4)	72.6	(9.8)	85.0	(19.4)	***
**Percentile range (°/s)**							
10th–90th	72.4	(13.8)	29.3	(5.0)	43.1	(9.8)	***
**Proportion of time (%)**							
<5°/s	19.4	(5.7)	39.1	(6.2)	−19.7	(2.5)	***
>90°/s	6.7	(3.0)	0.4	(0.3)	6.3	(2.8)	***
**Combined parameter**							
angle (−10° to 20°) AND vel < 5°/s	11.7	(3.8)	27.5	(5.8)	−15.8	(2.9)	***
Angle < 15° AND vel < 5 °/s	10.9	(3.9)	25.3	(6.6)	−14.3	(3.4)	***
Angle < 20° AND vel < 5 °/s	12.6	(4.1)	28.8	(6.2)	−16.2	(3.0)	***

***: *p* < 0.0001.

## Data Availability

The data that support the findings of this study are available on reasonable request from the corresponding author C.M.L. The data are not publicly available due to them containing information that could compromise research participant consent.
